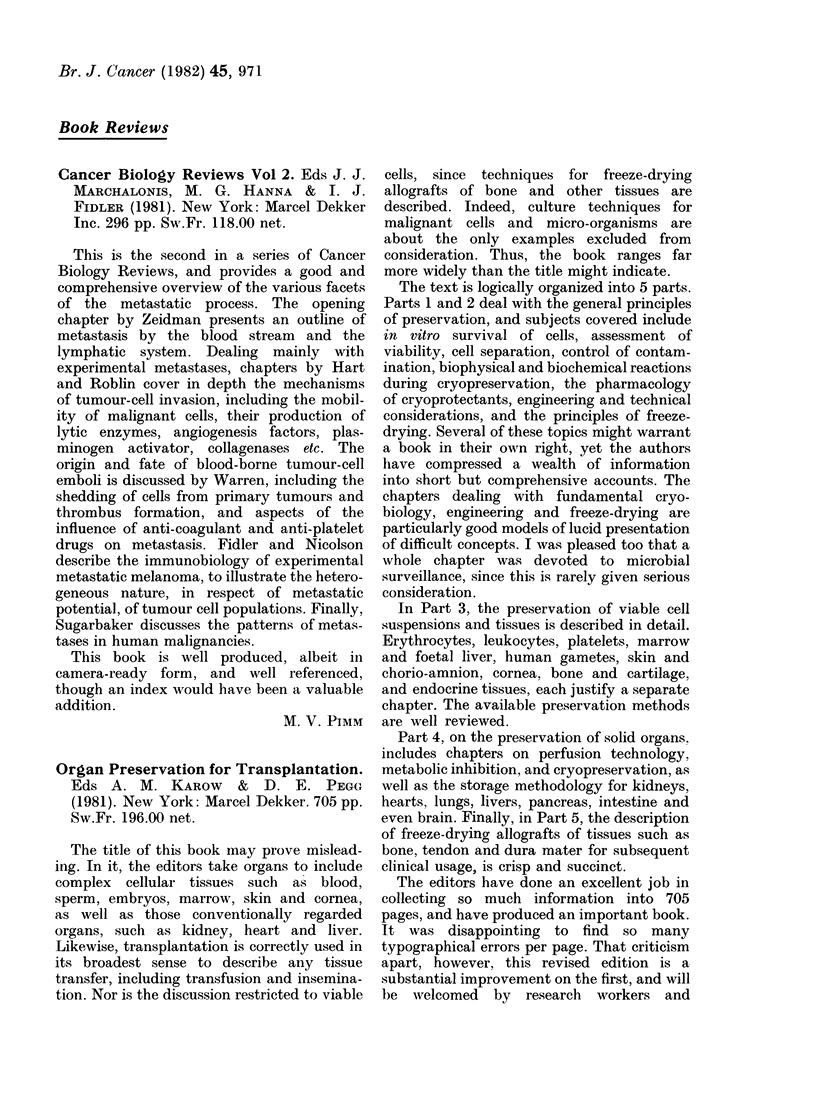# Cancer Biology Reviews Vol 2

**Published:** 1982-06

**Authors:** M. V. Pimm


### *Book Reviews*

**Cancer Biology Reviews Vol 2.** Eds J. J. MARCHALONIS, M. G. HANNA & I. J. FIDLER (1981). New York: Marcel Dekker Inc. 296 pp. Sw.Fr. 118.00 net.

This is the second in a series of Cancer Biology Reviews, and provides a good and comprehensive overview of the various facets of the metastatic process. The opening chapter by Zeidman presents an outline of metastasis by the blood stream and the lymphatic system. Dealing mainly with experimental metastases, chapters by Hart and Roblin cover in depth the mechanisms of tumour-cell invasion, including the mobility of malignant cells, their production of lytic enzymes, angiogenesis factors, plasminogen activator, collagenases *etc.* The origin and fate of blood-borne tumour-cell emboli is discussed by Warren, including the shedding of cells from primary tumours and thrombus formation, and aspects of the influence of anti-coagulant and anti-platelet drugs on metastasis. Fidler and Nicolson describe the immunobiology of experimental metastatic melanoma, to illustrate the heterogeneous nature, in respect of metastatic potential, of tumour cell populations. Finally, Sugarbaker discusses the patterns of metastases in human malignancies.

This book is well produced, albeit in camera-ready form, and well referenced, though an index would have been a valuable addition.

M. V. PIMM